# Role of sphingosine‐1‐phosphate receptors in vascular injury of inflammatory bowel disease

**DOI:** 10.1111/jcmm.16333

**Published:** 2021-02-17

**Authors:** Xuewen Wang, Shuhua Chen, Hong Xiang, Ziwei Liang, Hongwei Lu

**Affiliations:** ^1^ Center for Experimental Medicine the Third Xiangya Hospital of Central South University Changsha China; ^2^ Department of Cardiology the Third Xiangya Hospital of Central South University Changsha China; ^3^ Department of Biochemistry School of Life Sciences of Central South University Changsha China; ^4^ Department of Clinical laboratory Yueyang Hospital Affiliated to Hunan Normal University Yueyang China

**Keywords:** inflammatory bowel disease, lymphocyte, sphingosine‐1‐phosphate receptor, vascular injury

## Abstract

Sphingosine‐1‐phosphate receptors (S1PRs) have an impact on the intestinal inflammation of inflammatory bowel disease (IBD) by regulating lymphocyte migration and differentiation. S1PR modulators as an emerging therapeutic approach are being investigated for the treatment of IBD. However, the role of S1PRs in intestinal vessels has not drawn much attention. Intestinal vascular damage is one of the major pathophysiological features of IBD, characterized by increased vascular density and impaired barrier function. S1PRs have pleiotropic effects on vascular endothelial cells, including proliferation, migration, angiogenesis and barrier homeostasis. Mounting evidence shows that S1PRs are abnormally expressed on intestinal vascular endothelial cells in IBD. Unexpectedly, S1PR modulators may damage intestinal vasculature, for example increase intestinal bleeding; therefore, S1PRs are thought to be involved in the regulation of intestinal vascular function in IBD. However, little is understood about how S1PRs regulate intestinal vascular function and participate in the initiation and progression of IBD. In this review, we summarize the pathogenic role of S1PRs in and the underlying mechanisms behind the intestinal vascular injury in IBD in order for improving IBD practice including S1PR‐targeted therapies.

## 1 INTRODUCTION

IBD is a group of chronic, non‐specific and diffuse inflammatory diseases that occur in the gastrointestinal tract.^1^ IBD is manifested by recurrent attack, poor drug efficacy, high risk of developing related complications and high disability rate.^2^ The conventional treatments for IBD include glucocorticoid, salicylazosulfapyridine/5‐aminosalicylic acid and biological agents, which, however, have limited effectiveness because of low specificity, high prevalence of drug resistance and poor long‐term efficacy.^3^ As new immunomodulators, S1PR modulators could significantly reduce the localization of CD4 + T cells to the site of inflammation, regulate T‐lymphocyte differentiation and resultantly ameliorate inflammatory disorders. A phased series of clinical trials have been carried out to evaluate the efficacy of S1PR modulators in patients with IBD.^4^, ^5^, ^6^ However, because of the undesirable off‐target effects of these drugs, some unexpected vascular complications have been observed.

With an in‐depth understanding of the pathogenesis of IBD, the importance of blood vessel dysfunction in the intestinal mucosal lesions of IBD has received special attention.^7^, ^8^ It has been reported that IBD is frequently accompanied by endothelial injury, and endothelial progenitor cells, a marker for both endothelial repair and vascular healing, are significantly reduced in patients with IBD.^9^ Indeed, chronic inflammation can not only damage the structure of blood vessels but also cause their functional disorder.^10^, ^11^ More particularly, IBD‐associated functional changes in intestinal vasculature feature a diminished barrier function but increased angiogenesis.^12^, ^13^ Moreover, impaired vasodilation and aberrant leucocyte adhesion resulted from inflammation are also involved in the onset of IBD.^14^ Clearly, the expression of adhesion molecules, such as ICAM‐1, VCAM‐1, MAdCAM‐1 and E‐selectin secreted from vascular endothelial cells, is significantly increased in the intestinal microvessels in active IBD.^7^ However, the precise mechanism behind intestinal vascular injury in IBD is still unclear.

Sphingosine‐1‐phosphate (S1P) is an important signalling molecule produced during the metabolism of sphingomyelin, an abundant cell membrane component. By binding to S1P receptors (S1PRs) on the cell membrane, S1P exerts a multitude of regulatory effects including cell proliferation, migration, apoptosis, senescence, angiogenesis and barrier integrity, among others, and participates in the pathophysiology of various diseases.^15^, ^16^, ^17^ As important regulatory receptors during intestinal inflammation, S1PRs have been extensively examined for their association with lymphocyte migration. In recent years, S1PRs have been found abundantly expressed on vascular endothelial cells and regulate their function. Therefore, it is possible that manipulating the S1P/S1PR signalling may produce off‐target effects on intestinal vasculature while protecting against the immune disorder of IBD. We previously reported that increased expression of S1PR2 is closely related to the disruption of vascular endothelial cell barrier and angiogenesis, but inhibition of S1PR2 expression can reverse the vascular endothelial cell injury.^18^ In line with this finding, more studies support the involvement of S1PRs in the regulation of vascular endothelial cell barrier function, vascular density and leucocyte migration across vascular endothelium within inflame tissues.^19^, ^20^ However, little is known about the special importance of S1PRs in the intestinal vascular changes in IBD. Herein, we review the newly identified role and possible mechanism of S1PRs in intestinal vascular injury in IBD in order to help improve treatment options for patients with IBD.

## 2 INTESTINAL VASCULAR INJURY IN IBD

The aetiology of IBD is more complex than previously thought of being closely related to environment, microorganism, heredity and immunity.^21^, ^22^, ^23^ It seems that a combination of more than multifaceted factors mediates abnormal immune and inflammatory response in intestinal mucosa and promotes the occurrence of IBD.^24^, ^25^ Conventional drugs in the treatment of immune disorders are mainly anti‐inflammatory. However, their long‐term curative effects are poor due to low specificity and high prevalence of drug resistance. Therefore, seeking alternative therapeutic targets is gaining appreciation for improving IBD management. It has been found that vascular injury plays an important role in the pathogenesis of IBD. Pulse wave velocity, a measure of the severity of vascular injury, typically shows a significant increase in patients with IBD,^26^ suggesting that altered vascular structure and function occur in patients with IBD. In intestinal mucosa, microvessels are the major type of blood vessels, and in the submucosal microcirculation, arterioles and venules are dominant.^27^ As vascular endothelial cells line microvessels and small vessels that make them a key component of intestinal circulation, intestinal vascular endothelial cells may be required in the development of IBD.

Vascular endothelial cell injury, indicated by appreciable changes in endothelial cell function and/or structure, is a result of the imbalance of various regulatory substances.^28^, ^29^ IBD patients usually have a significantly reduced number of endothelial progenitor cells,^9^ suggesting that vascular endothelial cells are damaged in these patients. The functional damage of vascular endothelial cells in an inflammatory state primarily presents with impaired barrier function, vascular dysplasia, increased adhesion molecule expression, enhanced leucocyte permeability and dysregulated nitric oxide (NO) secretion. The major cause for intestinal vascular endothelial injury is the abnormal secretion of inflammatory mediators such as interleukin (IL)‐1, IL‐6, tumour necrosis factor (TNF)‐α, NO and vascular endothelial growth factor (VEGF) in intestinal mucosa.^30^, ^31^ The increased production of VEGF promotes intestinal vascular endothelial cell proliferation, whereas the increased secretion of other inflammatory mediators such as TNF‐α and IL‐6 can damage the structural integrity of endothelial cells, leading to vascular abnormality in barrier homeostasis and cell adhesion. Mechanistically, TNF‐α induces endothelial cell damage by activating NADPH oxidase (NOX), whose activation results in the increased production of superoxide.^32^ NO can dilate blood vessels and inhibit the expression of inflammatory cytokines and adhesion molecules; however, NO has been observed significantly decreased in the intestinal tissues of patients with IBD.^33^ TNF‐α can impair endothelium‐dependent vasodilation by affecting the utilization of NO.^34^ In addition, leucocyte adhesion and transmigration are also important steps in the inflammatory response.^35^, ^36^ It was found that the expression levels of adhesion molecules such as ICAM‐1, VCAM‐1, MAdCAM‐1 and E‐selectin in vascular endothelial cells are significantly up‐regulated in intestinal microvessels of IBD.^7^, ^37^, ^38^ Collectively, increased angiogenesis and impaired barrier function are the main causes of intestinal vascular injury in IBD.

## 3 S1PRs AND INTESTINAL ANGIOGENESIS IN IBD

Chronic inflammation is often associated with increased angiogenesis, which is a key contributor to the maintenance of chronic inflammation in gastrointestinal tract.^39^ Blood vessels are mainly composed of endothelial cells and parietal cells (vascular smooth muscle cells and pericytes).^40^ Angiogenesis normally originates from the sprouting of endothelial cells, followed by the destruction of blood vessel walls and the proliferation and migration of endothelial cells.^41^ The angiogenic process is tightly regulated by a wide array of angiogenic factors. For example, VEGF is a known potent angiogenic factor, which promotes endothelial cell proliferation, migration and angiogenesis.^42^ In inflamed IBD tissues, different types of inflammatory cells (eg macrophages and lymphocytes) can produce angiogenic factors and promote pathological angiogenesis.^13^ Compared with patients who are in remission, patients with active IBD have significantly higher levels of serum VEGF, indicative of VEGF as a promoter of abnormal angiogenesis in IBD.^43^ Hypoxia at the intestinal inflammatory sites has the ability to stimulate angiogenesis by inducing the production of VEGF, fibroblast growth factor and TNF‐α.^44^ In addition to VEGF, the expression levels of avβ3, a marker of angiogenesis, are increased in intestinal microvascular endothelial cells of IBD.^45^ Patients with IBD also show increased intestinal vascular density caused by vascular dysplasia. This increased intestinal vascular density has been confirmed in DSS‐induced experimental enteritis.^46^ Consistent with this, anti‐angiogenic therapy has been proved to be an effective approach for experimental enteritis. Small molecule inhibitors such as VEGF blocker can effectively reduce the production of inflammatory mediators and accordingly relieve intestinal inflammation induced by DSS.^47^

As a group of cell surface receptors, S1PRs are widely expressed on many types of cells, thus regulating cell proliferation, migration and angiogenesis.^48^, ^49^ S1PRs are highly expressed in vascular endothelial cells as well and therefore influence their function. The expression levels of S1PR1 were observed obviously increased in the intestinal mucosa of patients with ulcerative colitis and were related to the increased intestinal mucosal blood vessel density.^50^ In the mouse chronic IBD model, the expression of S1PR1 was also increased in intestinal submucosa and muscular microvessels.^51^ Further studies have shown that there is a significant change in vascular density in the inflamed tissues of myeloid S1PR1 knockout mice.^52^ Additionally, S1PR2 is another important driver of pathological angiogenesis in the process of inflammation, and S1PR2 antagonists can reverse the increase in vascular density caused by inflammation.^53^ Therefore, S1PRs are intimately associated with the increase in intestinal vascular density in IBD, but their downstream effectors are unclear.

**FIGURE 1:**
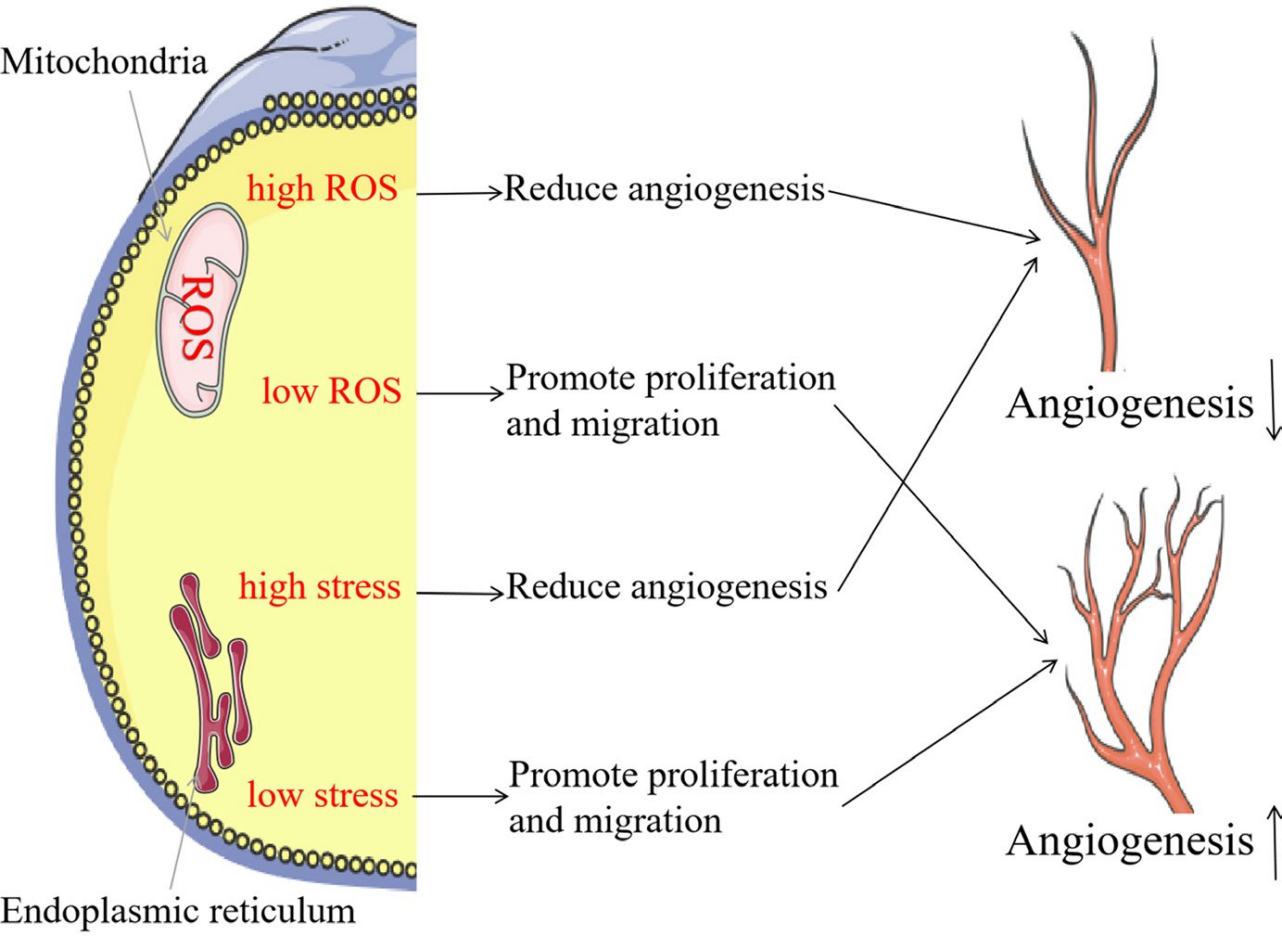
Low levels of ER stress/ROS stimulate angiogenesis, whereas high levels of ER stress/ROS inhibit angiogenesis

**FIGURE 2:**
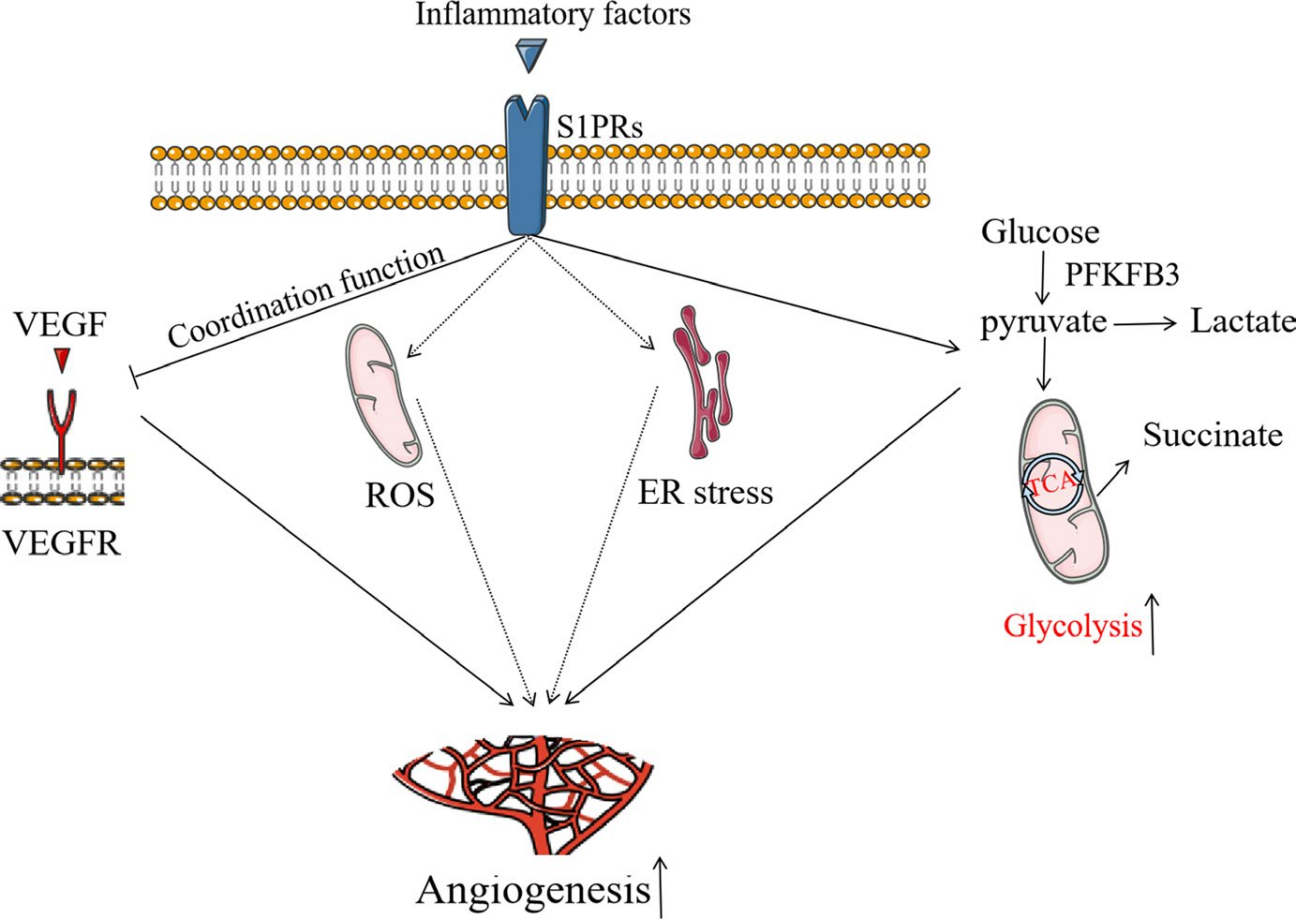
S1PRs may mediate intestinal angiogenesis of IBD by regulating ROS, ER stress, glycolysis and VEGF signal pathway

There are several important mechanisms governing angiogenic disorders, including oxidative stress, endoplasmic reticulum (ER) stress, abnormal glycolysis and activated VEGF signalling. In intestinal mucosa of patients with IBD, significantly increased ROS levels were seen.^54^ Under basic conditions, low levels of reactive oxygen species (ROS) act as messenger molecules to promote angiogenesis; however, high levels of ROS inhibit angiogenesis.^55^ ER stress levels were also increased significantly in inflammatory state.^56^ Like ROS, ER stress is closely related to angiogenesis; low levels of ER stress stimulate angiogenesis, whereas high levels of ER stress inhibit angiogenesis ^57^ (Figure 1). As it is difficult to quantitatively determine the levels of ROS and ER, the relationship between the ROS and ER levels and intestinal angiogenesis in IBD needs to be further evaluated. Moreover, glycolysis is known to be enhanced inflammation and S1P/S1PR signal pathway impacts glycolysis.^58^, ^59^ It has been demonstrated that glycolysis intermediates (eg lactate and succinate) and glycolytic enzymes (eg fructose‐6‐phosphate‐2 kinase and fructose‐2,6‐diphosphatase 3) are implicated in the regulation of angiogenesis.^60^ Besides this, S1PRs have a role in the regulation of VEGF‐mediated angiogenesis (co‐ordination function).^61^ Together, S1PRs may harbour broad effects on the intestinal angiogenesis of IBD by regulating ROS, ER stress, glycolysis, VEGF signal pathway and other processes, and hence, more understanding of their specific mechanisms is of great significance to the prevention and treatment of IBD (Figure 2).

## 4 S1PRs AND INTESTINAL VASCULAR BARRIER FUNCTION IN IBD

Vascular endothelium is a highly specialized monolayer epithelium that lines the inner surface of blood vessels and functions in regulating the transport of substances and leucocyte transmigration across blood vessels.^62^, ^63^ Under physiological conditions, endothelial cells protect against excessive vascular permeability and leucocyte adhesion.^64^ During inflammation, vascular endothelial response leads to vasodilation and increased permeability, followed by structural changes such as endothelial cell proliferation and vascular remodelling.^65^, ^66^ It has been confirmed that the intestinal vascular barrier function is impaired in the animal model of ulcerative colitis.^7^ The disruption of vascular barrier function can be attributed to the elevated levels of endothelial cell oxidative stress and ER stress as well as other subcellular ultrastructural abnormalities. Oxidative stress in intestinal vascular vessels of IBD patients can damage vascular endothelium and increase microvascular permeability.^12^ Moreover, inflammation exacerbates such endothelial damage by increasing ROS production and enhancing ER‐mitochondria contact.^67^, ^68^ ER stress impairs vascular barrier function by disturbing the endothelial cell‐to‐cell junctions.^69^, ^70^ ER stress is increased in endothelial cells under inflammatory conditions, and inhibition of ER stress will reverse the impairment of barrier function.^71^, ^72^ Therefore, endothelial cell oxidative stress and ER stress are two key culprits in the dysfunction of intestinal vascular barriers in IBD, but the precise mechanisms that link these aspects need further clarification.

**FIGURE 3:**
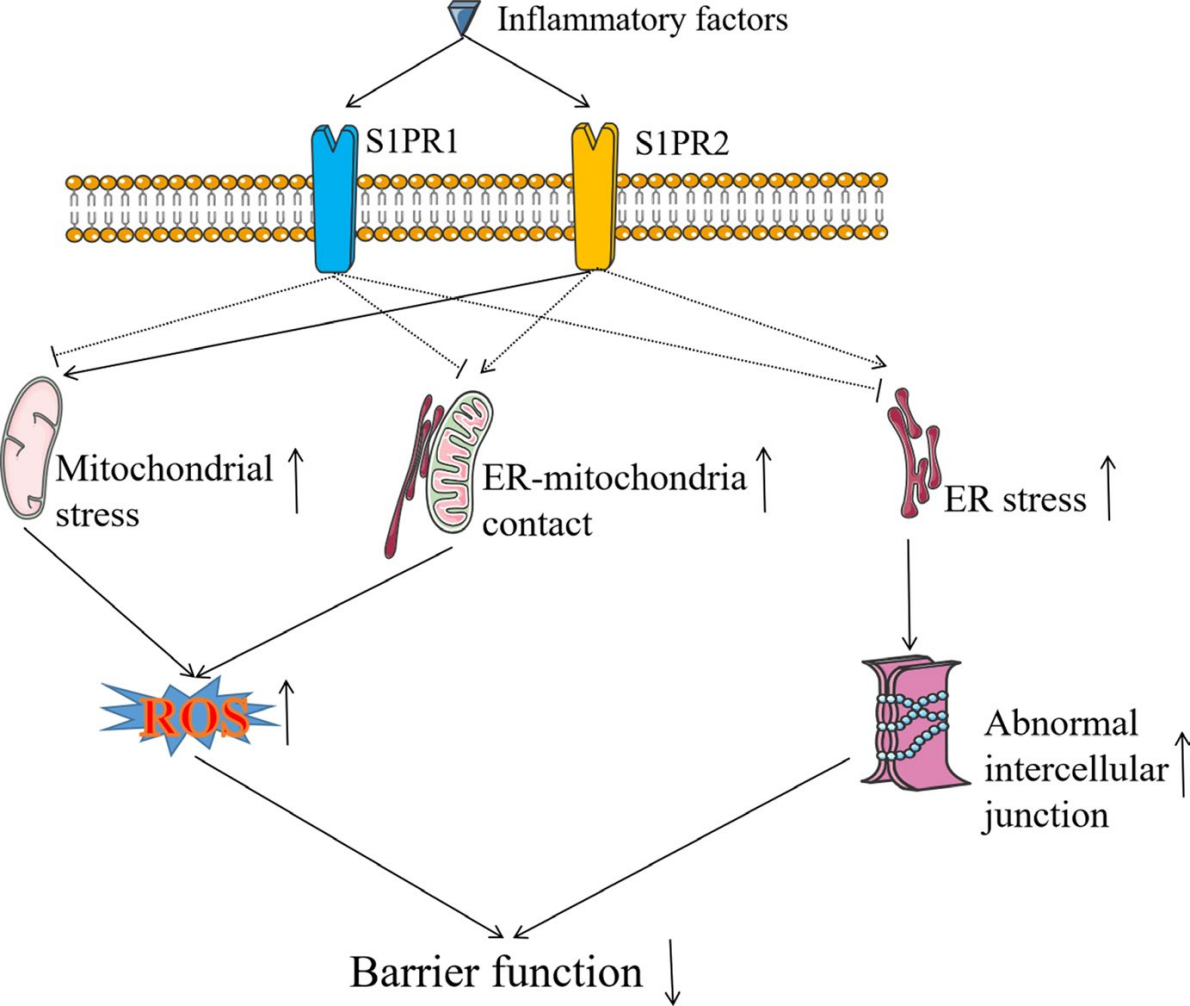
S1PRs may affect the intestinal vascular barrier function of IBD by regulating mitochondrial stress, ER stress or ER‐mitochondria contact

S1PRs may represent an important mechanism in the regulation of vascular barrier function.^73^ S1PR1‐3 has been proven to be responsible for this complex regulatory mechanism. S1PR1 basically maintains the vascular barrier function. This was demonstrated by deleting S1PR1 gene in IBD that resulted in increased intestinal vascular permeability, indicated by increased bleeding in experimental enteritis.^50^ On the contrary, S1PR2 contributes to the impairment of vascular barrier function. An increased expression of S1PR2 has been observed in endothelial cells in the state of inflammation, which resulted in the impairment of endothelial cell barrier function.^74^ Similarly, S1PR3 is a player in the regulation of vascular barrier function. Either S1PR2 gene knockout or decreased expression of S1PR3 can reverse the impairment of vascular barrier function in inflammatory state.^75^ The same studies showed that the expression levels of both S1PR1 and S1PR2 in vascular endothelial cells were increased under the condition of IBD or inflammation, indicating that these two S1PRs with opposite effects on vascular barrier function may control each other's regulatory magnitude. Although these studies have highlighted the role of S1PR2 in the impairment of intestinal vascular barrier function in IBD, we have reported that S1PR2 antagonists can reduce the production of ROS in endothelial cells.^76^ The increase in ROS is mediated by mitochondrial stress or ER‐mitochondrial contact. In addition, ER stress is closely related to abnormal intercellular junctions. Therefore, S1PR2 may affect intestinal vascular barrier function in IBD by increasing oxidative stress, ER stress or ER‐mitochondrial contact (Figure 3).

## 5 S1PRs AND LEUCOCYTE MIGRATION ACROSS VASCULAR ENDOTHELIUM IN IBD

The extravasation of leucocyte from the bloodstream into inflamed tissues is a hallmark of inflammatory response, which involves the interaction between immune cells and vascular endothelial cells.^77^, ^78^ Leucocyte migration across vascular endothelium proceeds in several steps including rolling, activation, adhesion and transport. Adhesion is the key step in determining whether leucocytes migrate to sites of inflammation. Inflammatory mediators signal vascular endothelial cells to express adhesion molecules and chemotactic cytokines to promote the adhesion of leucocytes to vascular endothelial cells. The adhesion ability of leucocytes and the expression of adhesion molecules such as ICAM‐1 and VCAM‐1 by intestinal microvessels of patients with IBD are enhanced.^13^, ^63^ The expression of fractalkine, a CX3C chemokine, is also significantly up‐regulated in endothelial cells of patients with IBD.^79^ Intriguingly, there is a complex interplay of immune cells and angiogenesis in intestinal inflammation. For instance, VEGF‐A can induce cAMP production and activate the cAMP signalling in intestinal vascular endothelial cells to promote leucocyte adhesion.^80^ On the other hand, the increased leucocyte migration across vascular endothelium results in increased intestinal inflammation, thus creating a positive feedback loop that exacerbates inflammatory response.^4^ Therefore, blockade of leucocyte adhesion and migration across vascular endothelium holds the key to alleviating intestinal inflammation in IBD and is becoming important for an effective IBD therapeutic tactic.

As important receptors on leucocytes, S1PRs participate in the regulation of leucocyte transport and differentiation.^4^, ^81^ Emerging studies have shown evidence of the active involvement of S1PRs in the occurrence and development of the intestinal inflammation by regulating endothelial function and its interaction with immune cells. It has been found that S1PR1 is expressed in intestinal lymphocytes and vascular endothelial cells of IBD. Because of this, S1PR1 modulators have been used to reduce lymphocytes in intestinal tissue by inhibiting lymphocyte migration, thereby alleviating intestinal inflammation.^51^ In addition, S1PR1 modulators inhibit the adhesion of monocytes to endothelial cells by reducing the expression of VCAM‐1 in vascular endothelial cells.^82^ The expression of S1PR2 in vascular endothelial cells is increased in inflammatory state.^74^ It has been found that S1PR1 and S1PR4 differentially regulate T cell migration and adhesion, whereas S1PR2 promotes T cell migration across endothelial cells by increasing endothelial cell permeability and VCAM‐1 expression.^83^ Therefore, S1PRs take part in intestinal inflammation of IBD by regulating leucocyte migration across vascular endothelial cells, and S1PR modulators have an inhibitory effect on intestinal inflammation.

## 6 S1PRs AND ENDOTHELIAL NITRIC OXIDE SYNTHASE IN IBD

Endothelial nitric oxide synthase (eNOS) is one of the main modulators of the vascular function by promoting NO secretion, with the latter causing vasorelaxation and inhibiting the expression of cytokines and adhesion molecules. Therefore, eNOS‐mediated NO release from vascular endothelial cells is important in the regulation of vascular inflammation. There is evidence in support of eNOS as a key molecule in colitis by regulating the expression of adhesion molecules (eg AdCAM‐1).^84^ Considering the protective role of eNOS in experimental colitis, it was not surprised to observe a significant decrease in eNOS expression in the intestinal microvessels of IBD patients.^85^ Moreover, decreased eNOS in endothelial cells is considered a possible reason for the thickening of arteries and the increased number of capillaries in IBD intestinal mucosa.^85^ It has been observed that NO deficiency in intestinal microvascular endothelial cells aggravates experimental colitis.^86^ Also, decreased NO affects the dilation for microvessels, which further decreases arterial perfusion and delays wound healing.^87^ Nevertheless, excessive release of NO may have an opposite effect on vascular tone. Previous studies have demonstrated that excessive NO produced by the inducible isoform of NOS contributes to intestinal inflammation in IBD, though eNOS produces NO with a far less amount, which in fact has a physiological role in the gut.^88^, ^89^ Therefore, both the source and quantity of NO matter; they decide how intestinal inflammation is controlled in IBD.

**FIGURE 4:**
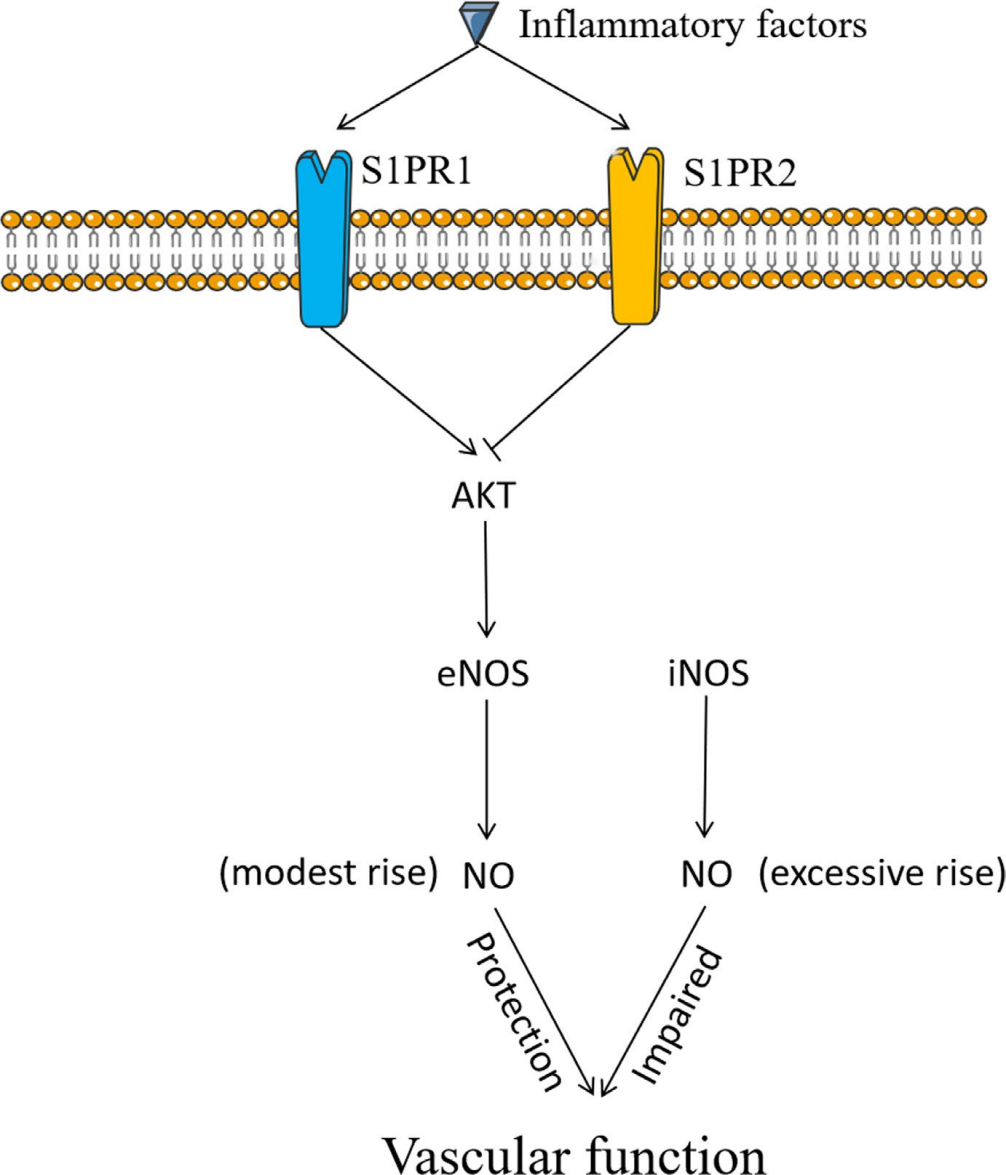
S1PRs may regulate vascular function by regulating the source and quantity of eNOS

S1PRs play a vital important role in eNOS regulation. They regulate eNOS by activating protein kinase B (AKT), which is involved in the regulation of endothelial cell migration and angiogenesis.^90^ However, the overexpression of eNOS may cause an injury under pathological conditions, for example, promote angiogenesis and damage barrier function. It has been proposed that S1PR2 activation in vascular endothelial cells enhances the expression of eNOS, and thus, antagonizing S1PR2 may be a new approach for the treatment of pathological angiogenesis.^53^ On the other hand, in anaphylactic shock S1PR2 inhibits excessive eNOS‐mediated endothelial barrier dysfunction by decreasing AKT.^91^ Therefore, more evidence is needed to understand the regulation of intestinal eNOS by S1PRs in IBD. Considering the significant role of eNOS in the regulation of vascular function and inflammation, it is necessary to determine the regulatory roles of different sources and quantities of eNOS in various disease states (Figure 4). Collectively, It is of great significance to clarify the regulatory effects of S1PRs on intestinal eNOS in IBD.

## 7 EFFECTS OF S1PR REGULATORS ON INTESTINAL VESSELS OF IBD

**FIGURE 5:**
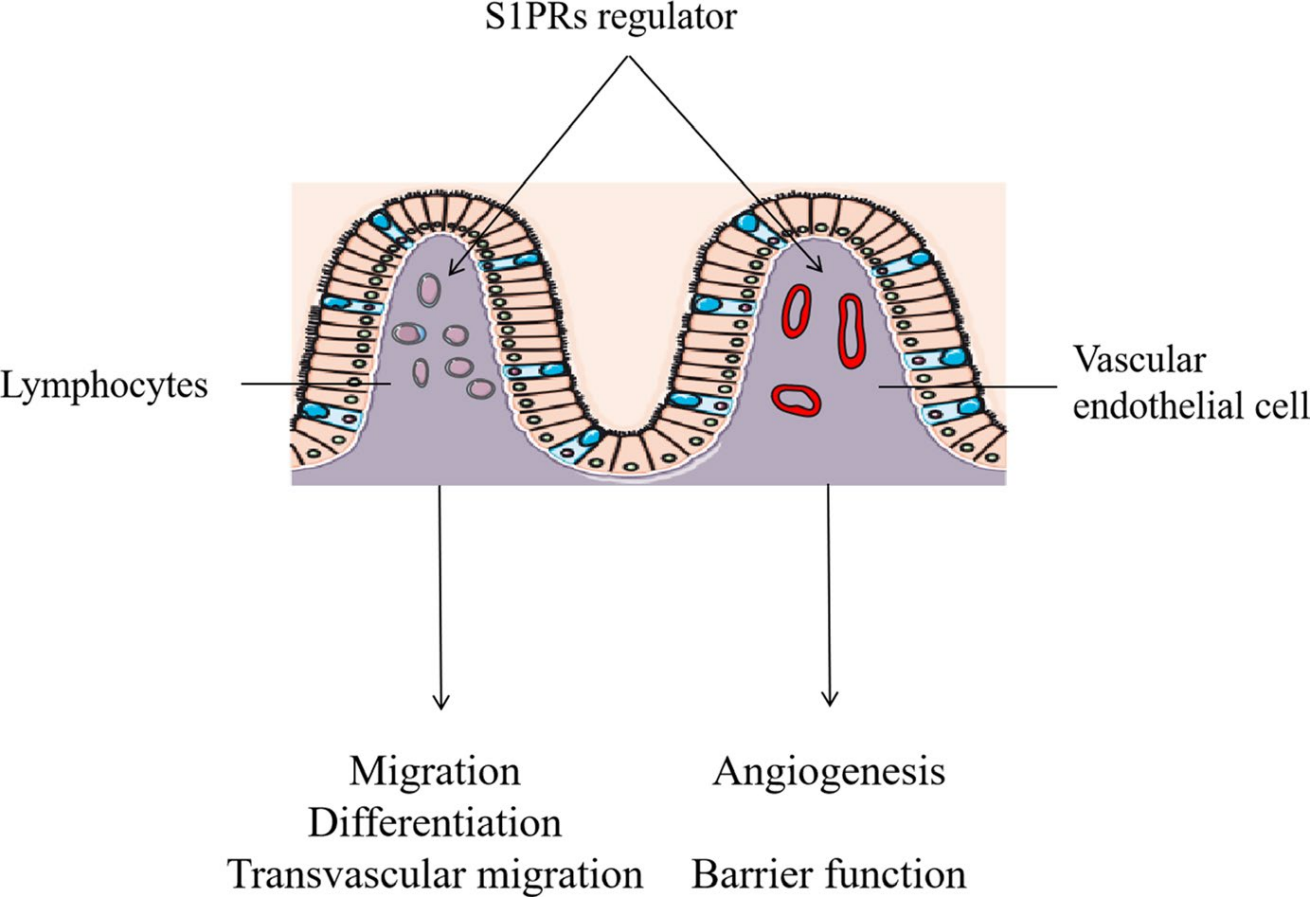
S1PR regulators have influences not only on lymphocytes but also on vascular endothelial cells

The pathological process of IBD ranges from active stage to remission stage, and an effective therapeutic strategy is to control the active phase of the illness.^92^, ^93^ The injury of intestinal vascular endothelial cells during the active stage is chiefly because of the sustained inflammation and oxidative stress. Therefore, anti‐inflammatory and antioxidant therapy on IBD is very important. For example, the anti‐inflammatory treatment using TNF‐α antagonists could largely decrease the levels of plasma biomarkers of endothelial dysfunction in patients with IBD.^14^ Targeting the S1P/S1PR axis is an efficacious option for IBD, leading to the development of S1PR modulators in recent years. As a new class of immunomodulators, S1PR modulators can improve intestinal inflammation by reducing the intestinal infiltration of lymphocytes and inhibiting T cell differentiation in patients with IBD.^4^, ^51^, ^94^ However, other studies have found that S1PR modulators may have some intestinal vascular effects while suppressing immune responses (Figure 5). Karuppuchamy *et al* suggest that S1PR modulators may have unwanted effects on intestinal vascular barrier function.^51^ Montrose *et al* found that the genetic deletion of S1PR1 increased intestinal vascular permeability and bleeding in experimental colitis.^50^ In addition, FTY720 produces anti‐inflammatory effects by inhibiting the interaction between immune cells and vascular endothelial cells.^95^ Although S1PR modulators have unexpected side effects on intestinal vessels and different subtypes of receptor modulators have distinct effects, the underlying mechanisms are not completely clear.

At present, the S1PR modulators used in IBD mainly include non‐specific regulators such as FTY720 and specific regulators such as ozanimod (targeting S1PR1 and S1PR5), etrasimod (targeting S1PR1) and amiselimod (targeting S1PR1 and S1PR5), which have entered phase II or III clinical trials or approved for IBD. The expression levels of S1PR1, S1PR2 and S1PR4 are significantly higher in the intestinal mucosa of IBD, and S1PR1 is primarily present on leucocytes and vascular endothelial cells.^51^, ^96^, ^97^ As the up‐regulated S1PR1 in lymphocytes mediates immune abnormalities at sites of inflammation, these novel regulators mainly exert influence on the immunomodulatory function of S1PR1. For example, the S1PR agonist FTY720 first activates S1PR1 on the cell surface through a high‐affinity binding, but then causes the down‐regulation of S1PR1, thus preventing lymphocyte infiltration.^98^ This regulator, which first activates the surface receptor and then downregulates the receptor, is often called a reverse agonist. Conventional S1PR inhibitors mostly work via a direct inhibition of the expression of cell surface receptors, but the S1PR reverse agonist acts through a process named receptor endocytosis. Under physiological conditions, the endocytosed S1PRs return to the cell surface through S1P lyase–mediated mechanism, but under continuous stimulation by the reverse agonist, endocytosed S1PRs are degraded by the proteasome, resulting in complete inactivation of S1PR1.^99^ As mentioned earlier, in the intestinal tract of IBD, S1PR1 is mainly involved in the regulation of angiogenesis and barrier function, whereas S1PR2 is essential for the regulation of barrier function. At present, the most reported intestinal vascular effects of S1PR regulators are substantially attributable to the potential impact of barrier function mediated by the down‐regulation of S1PR1. However, whether the altered blood vessel density is related to S1PR1 regulators or the barrier dysfunction related to S1PR2 regulators remains unclear. Therefore, it is necessary to explore the effects of S1PR modulators on intestinal vascular function, which may help improve or develop new S1PR modulators.

## 8 CONCLUSION

S1PRs are a group of receptors expressed on the surface of various cell types, and different subtypes have distinct regulatory functions. In this review, we depicted the negative role of S1PRs in intestinal vascular injury in IBD and their possible mechanism. So far, the exact mechanisms behind the biological regulation of S1PRs in the intestinal vascular disease are rather limited. Because of their vital role in lymphocytes, S1PRs have become an important target for immunomodulatory therapy. The application of S1PR modulators in patients with IBD is a focus of this review. A series of clinical trials have, however, disclosed that S1PR modulators may cause intestinal vascular effects. For example, S1PR1 modulators down‐regulate S1PR1 of vascular endothelial cells while inhibiting S1PR1 of lymphocytes, resulting in a decline in vascular barrier function. So, how to overcome the unwelcome side effects of S1PR modulators is still a challenge. S1PRs seem to have a widespread impact on intestinal vascular biology, such as vascular density, barrier function and ultrastructural changes in vascular endothelial cells (ie ER stress, mitochondrial stress, ER‐mitochondria contact). Further studies of the role of S1PRs in intestinal vessels will be of benefit in improving IBD care.

## CONFLICT OF INTEREST

The authors confirm that there are no conflicts of interest.

## AUTHOR CONTRIBUTION

**Xuewen Wang:** Conceptualization (equal); Investigation (lead); Resources (equal); Writing‐review & editing (lead). **Shuhua Chen:** Funding acquisition (lead); Supervision (equal). **Hong Xiang:** Investigation (supporting); Resources (equal). **Ziwei Liang:** Investigation (supporting); Resources (equal). **Hongwei Lu:** Conceptualization (lead); Funding acquisition (lead); Supervision (equal); Writing‐review & editing (equal).
